# Osteophytes

**Published:** 1873-05

**Authors:** 


### OSTEOPHYTES.

Sometimes, in consequence of diseases of the teeth, and, particularly, of chronic inflammation of the root membrane, an irritation is induced in the maxillary periosteum, which is attended by its tumefaction and sensitiveness, occasions consecutive œdema, and not infrequently gives rise to the formation of an osteophyte of a fine spongy texture.

If a series of macerated jaws, which have contained carious teeth, be examined, several of these growths will usually be found, in the form of cribriform, unyielding lamellæ, generally adherent to the facial wall, more commonly to that of the lower jaw, and distinguished by a quite bright color. When peeled off and examined in thin sections they prove to be young osseous substance. They are located, almost always, upon the posterior segment of the jaw, are of very variable extent and present very irregular outlines ; their thickness varies from one-quarter to one millimetre. They occur in isolated patches, and occasionally spread over the entire surface of the maxillary wall.

The period of puberty, together with the retarded eruption of the wisdom teeth, appear to be especially favorable for the development of osteophytes, and, when associated with an incomplete growth of the jaws, they are to be eonnected with unusual succulence of the periosteum.

